# Cyclic di-AMP signaling and regulation in *Staphylococcus aureus*

**DOI:** 10.1128/msphere.00019-26

**Published:** 2026-07-31

**Authors:** Seonmin Lee, Ji-Hoon Kim, Taegwan Yun, Wonsik Lee

**Affiliations:** 1 Department of Pharmacy, School of Pharmacy, Sungkyunkwan University 105934, Suwon, Republic of Korea; 2 Department of MetaBioHealth, Sungkyunkwan University 35017, Suwon, Republic of Korea; 3 Department of Biopharmaceutical Convergence, Sungkyunkwan University 65666, Suwon, Republic of Korea; Shenzhen Institute of Synthetic Biology, Shenzhen, China

**Keywords:** cyclic di-AMP, bacterial signaling molecule, bacterial physiology, antibiotic resistance, *Staphylococcus aureus*

## Abstract

Cyclic di-AMP (c-di-AMP) is an essential bacterial second messenger that coordinates multiple cellular processes and is closely linked to central physiology in many bacteria. In *Staphylococcus aureus*, both depletion and excessive accumulation of c-di-AMP impair bacterial fitness, indicating that this signaling pathway must be tightly regulated. Recent studies have identified c-di-AMP receptor proteins in *S. aureus*, revealing how c-di-AMP maintains osmotic balance through ion transport and influences cell envelope function and stress adaptation. These regulatory effects also influence bacterial growth and cell size, and are linked to biofilm formation and β-lactam resistance. Elevated c-di-AMP levels can alter peptidoglycan architecture and reduce autolysis, thereby increasing β-lactam resistance, whereas reduced c-di-AMP levels can increase β-lactam susceptibility. This review summarizes current understanding of c-di-AMP synthesis, degradation, and receptor-mediated signaling in *S. aureus*. We also discuss emerging approaches to perturbing intracellular c-di-AMP balance as potential antimicrobial strategies.

## INTRODUCTION

Bacteria are continuously exposed to diverse environmental changes and stresses. They are especially susceptible to such stimuli and therefore rely on efficient regulatory systems to sense and adapt rapidly for survival. Bacteria have evolved intricate signal transduction systems to sense environmental changes and coordinate cellular responses. Among these, second messengers enable the regulation of multiple cellular processes through rapid and integrated signaling. Major bacterial second messengers include cyclic AMP (cAMP), cyclic di-GMP (c-di-GMP), and cyclic di-AMP (c-di-AMP) (1). These signaling molecules function by binding directly to target proteins or RNAs, thereby modulating their activity to regulate cellular physiology. c-di-AMP has recently emerged as an essential second messenger in several bacteria, influencing stress adaptation, antibiotic resistance, and virulence.

c-di-AMP signaling is widespread in the bacterial domain and even present in some archaea, but its physiological roles can vary across species. c-di-AMP is produced mostly by many gram-positive bacteria, such as *Staphylococcus aureus*, *Streptococcus pneumoniae*, *Listeria monocytogenes*, and *Mycobacterium tuberculosis*, and by some gram-negative bacteria and archaea. Although the proteins involved in c-di-AMP synthesis and signaling differ among organisms, control of ion and osmotic homeostasis is a broadly conserved function. Through this activity, c-di-AMP helps maintain intracellular solute balance and protects cells from changes in environmental osmolarity. Its cellular concentration must therefore be tightly controlled, as either depletion or excessive accumulation can disrupt growth and cell envelope integrity. This conservation across phylogenetically diverse bacteria suggests that c-di-AMP represents an evolutionarily important signaling system that has been adapted to different physiological requirements. Of these pathogens, *S. aureus* is a major human pathogen that colonizes the skin and mucosa and causes diseases ranging from superficial infections to life-threatening conditions such as bacteremia and pneumonia (2). For pathogenic bacteria, rapid adaptation to host environments is essential for infection, and in *S. aureus*, c-di-AMP plays a critical role in these adaptive responses by coordinating multiple cellular processes (3). In this review, we provide a comprehensive overview of c-di-AMP signaling in *S. aureus*, with a focus on its synthesis, degradation, receptor proteins, and roles in bacterial physiology and pathogenesis. We further discuss emerging themes, including the therapeutic potential of targeting c-di-AMP pathways, the interplay of c-di-AMP with host immunity, and comparative perspectives from other species to contextualize *S. aureus* c-di-AMP signaling in the broader microbiological landscape.

## c-di-AMP SIGNALING IN BACTERIA

**Fig 1:**
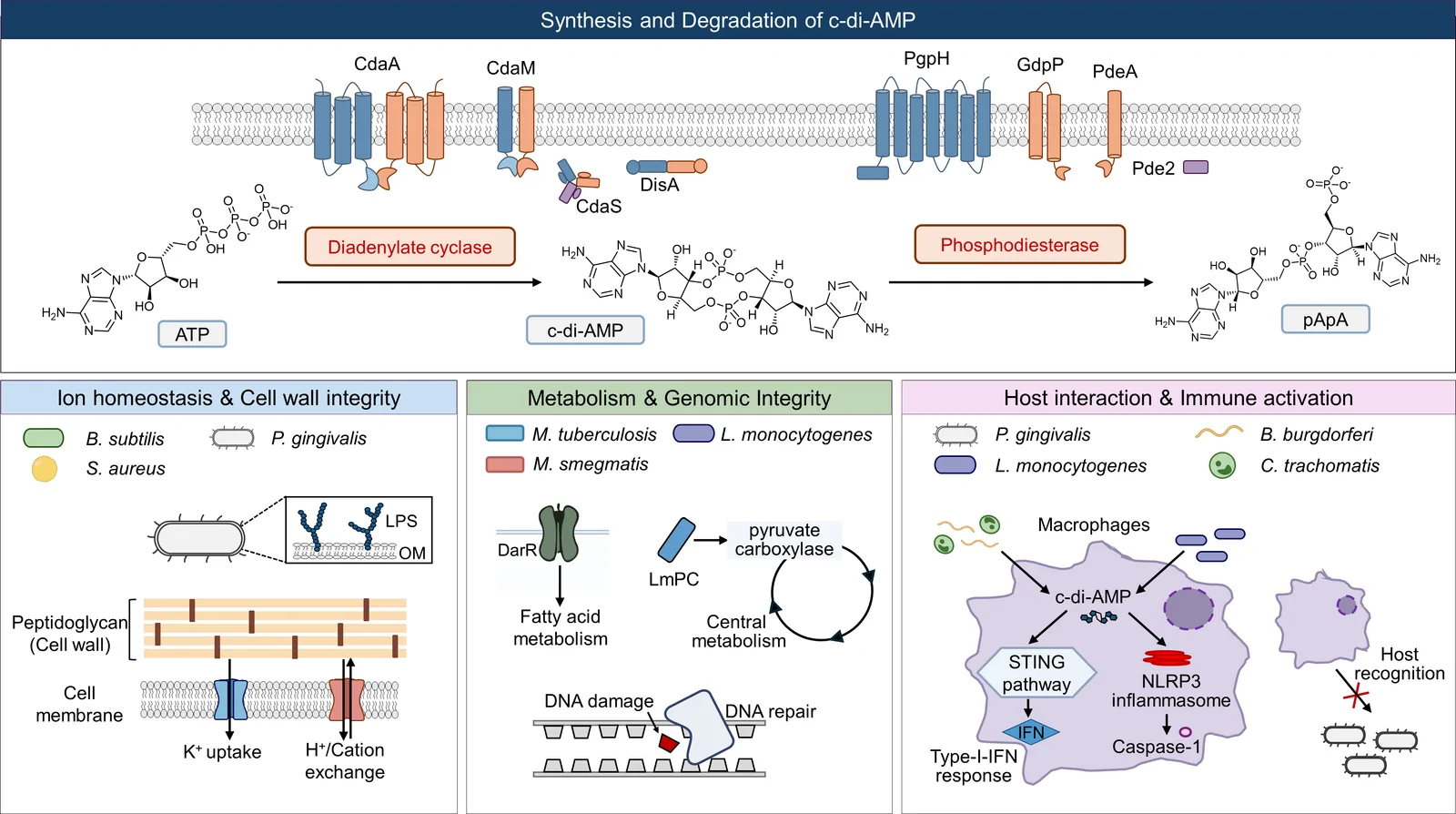
Metabolism and functional axes of bacterial c-di-AMP. (Top) Synthesis of c-di-AMP by DACs (CdaA, DisA, CdaS, and CdaM) and degradation by PDEs (GdpP, PgpH, PdeA, and Pde2). (Bottom left) Maintenance of osmotic homeostasis and cell wall integrity through regulation of K^+^ transport and envelope biogenesis. (Bottom middle) Integration with central metabolism, fatty acid biosynthesis, and DNA repair for cellular stress adaptation. (Bottom right) Modulation of host innate immunity via secreted c-di-AMP and activation of inflammasome pathways.

c-di-AMP is synthesized from two molecules of ATP or ADP by cyclase domain-containing proteins known as diadenylate cyclases (DACs) (4, 5). Four classes of DAC enzymes have been identified in bacteria: DisA, CdaA, CdaS, and CdaM. Among these, CdaA is the most widely conserved diadenylate cyclase and is the only DAC in many gram-positive bacteria. *S. aureus* also encodes a single enzyme of the CdaA class, known as DacA (Fig. 1). As a global regulator, c-di-AMP is closely linked to osmotic homeostasis and cell envelope stability and therefore is often essential for optimal growth. Imbalanced cellular c-di-AMP alters stress tolerance, leading to either resistance or sensitization depending on bacterial physiology and environmental conditions. These trade-offs are organism-dependent, and regulatory systems vary across species, highlighting the evolutionary plasticity of c-di-AMP signaling and its integration with other global regulators such as (p)ppGpp. c-di-AMP signaling is more common in gram-positive bacteria, which encode genes for its production and turnover.

In *Bacillus subtilis*, c-di-AMP coordinates cell envelope integrity and osmotic balance. It is produced by three DACs (DisA, CdaA, and CdaS) and degraded by two PDEs (GdpP and PgpH) (6, 7). DisA contributes to genome integrity, CdaA to cell wall and K^+^ homeostasis, and CdaS to spore germination (6–8). Reduced c-di-AMP compromises cell envelope integrity and increases susceptibility to β-lactam antibiotics, whereas elevated c-di-AMP can increase β-lactam antibiotic resistance but causes potassium transport defects and osmotic sensitivity (9–11). (p)ppGpp further links nutrient status to c-di-AMP signaling by inhibiting PDEs during starvation. In *Listeria monocytogenes*, this balance is tightly coupled to host adaptation (12, 13). *L. monocytogenes* encodes one cyclase (DacA) and two PDEs (PdeA and PgpH), and c-di-AMP is essential for growth and acid stress resistance (5). Elevated c-di-AMP impairs intracellular growth and attenuates virulence, whereas reduced levels increase sensitivity to cell wall-targeting antibiotics. *Listeria* also secretes c-di-AMP during infection, linking bacterial signaling to host immune activation. c-di-AMP regulates metabolic enzymes such as pyruvate carboxylase (LmPC), connecting nucleotide signaling to central metabolism (14). These features may partly reflect pathogen lifestyle differences: extracellular bacteria such as *S. aureus* tolerate higher c-di-AMP, whereas intracellular pathogens such as *Listeria* maintain lower levels to limit host immune activation.

In *Streptococcus* species, c-di-AMP regulation produces diverse phenotypes in biofilm formation and virulence. *Streptococcus pneumoniae* encodes one CdaA and two PDEs, and c-di-AMP controls K^+^ uptake through CabP. PDE mutants show cell separation defects, indicating altered envelope biogenesis (15, 16). In *Streptococcus mutans*, deletion of *cdaA* increases oxidative stress sensitivity but enhances polysaccharide production, whereas PDE deletion increases biofilm formation (17). In *Streptococcus pyogenes*, c-di-AMP regulates virulence gene expression, where *gdpP* deletion reduces SpeB production and virulence while increasing antibiotic tolerance (18, 19). In mycobacteria, which possess a lipid-rich cell envelope, c-di-AMP contributes to envelope regulation through distinct mechanisms. *Mycobacterium tuberculosis* encodes the DisA-family cyclase Rv3586, also called DisA or DacA, and the DHH/DHHA1 phosphodiesterase Rv2837c, also called CnpB (20). Although not strictly essential *in vitro*, c-di-AMP is required for DNA repair and cell envelope regulation, and PDE mutants show reduced virulence *in vivo*. In *Mycobacterium smegmatis*, the c-di-AMP receptor DarR regulates ion transport and lipid homeostasis, and its deletion alters cell size and fatty acid metabolism (21). CdaM represents a recently identified DAC family characterized in *Mycoplasma pneumoniae*, where it is essential for growth. The c-di-AMP produced by CdaM regulates potassium homeostasis by binding to KtrC and inhibiting the low-affinity potassium transporter KtrCD (22).

Although less common in gram-negative bacteria, c-di-AMP also contributes to host interaction and envelope regulation. In *Chlamydia trachomatis*, c-di-AMP is secreted into the host cytosol and activates STING-dependent interferon responses (23). This response can restrict bacterial growth, although *Chlamydia* may exploit it to modulate the host environment. In other gram-negative pathogens, c-di-AMP can influence envelope architecture and immune recognition. *Porphyromonas gingivalis* relies on c-di-AMP for growth, coordinating outer membrane integrity and biofilm formation, and alterations in c-di-AMP affect membrane integrity and LPS structure, altering host recognition (24). In *Borrelia burgdorferi,* which lacks LPS, c-di-AMP is secreted extracellularly and functions as an alternative inflammatory signal. Macrophages respond by producing interferons and other immune responses, indicating that c-di-AMP can function alongside classical PAMPs (25).

## c-di-AMP PRODUCTION BY DacA IN *S. AUREUS*

**Fig 2:**
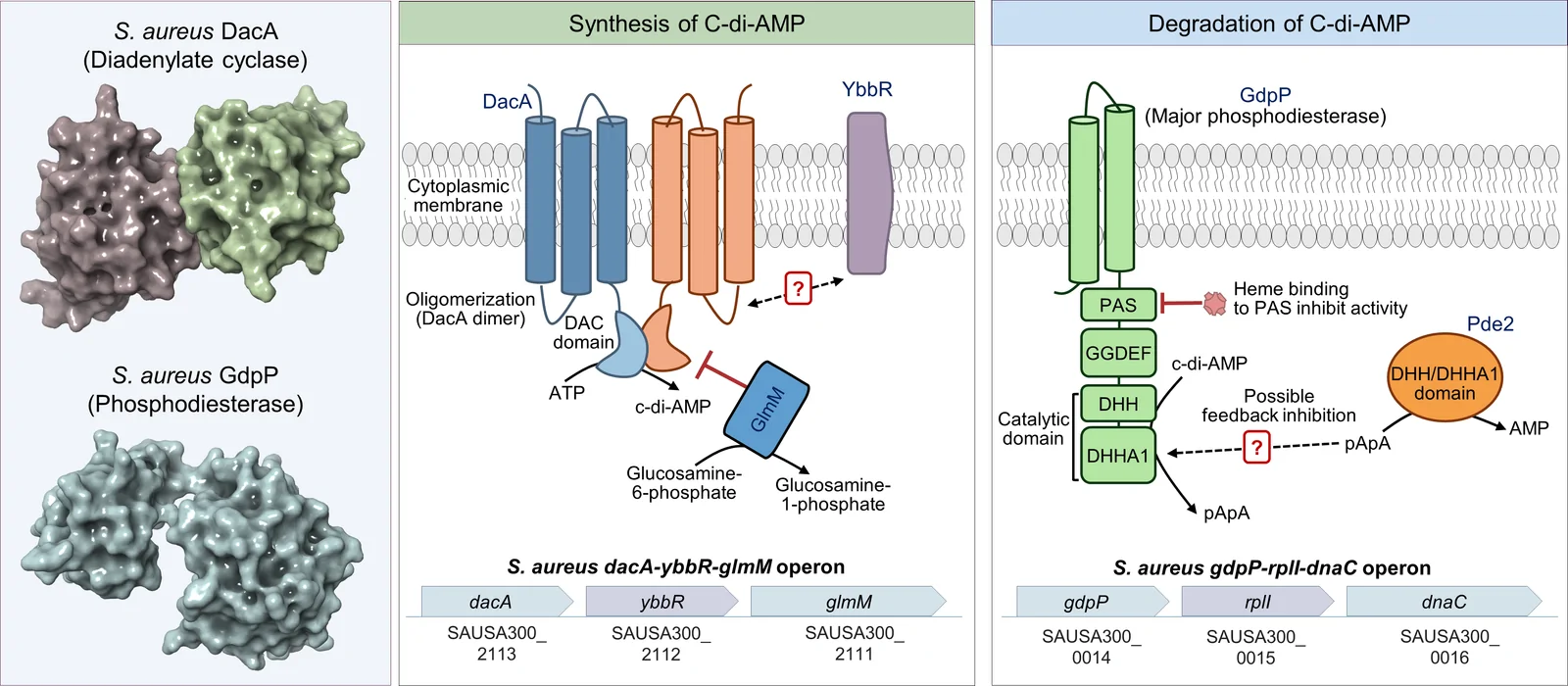
Homeostatic regulation of c-di-AMP in *Staphylococcus aureus*. (Left) Representative crystal structures of the key c-di-AMP metabolic enzymes in *S. aureus*: the diadenylate cyclase DacA (PDB ID: 6GYX) and the catalytic domain of phosphodiesterase GdpP (PDB ID: 5XSI). (Middle) DacA, the sole cyclase in *S. aureus*, requires oligomer formation for enzymatic activity. GlmM directly binds to the DAC domain of DacA, sterically hindering oligomerization and suppressing c-di-AMP production. (Right) The degradation of c-di-AMP occurs in two distinct stages: first, the primary phosphodiesterase GdpP, which is anchored to the cell membrane, hydrolyzes c-di-AMP into the intermediate pApA. GdpP activity is further modulated by its PAS domain, which senses cellular cues such as heme levels. Subsequently, the cytosolic enzyme Pde2 specifically targets the resulting pApA and hydrolyzes it into two molecules of AMP.

In *S. aureus*, c-di-AMP is synthesized by DacA, a membrane-bound diadenylate cyclase with three N-terminal transmembrane helices and a cytosolic DAC domain (Fig. 2). The *dacA* gene is at the beginning of an operon followed by *ybbR*, also known as *cdaR*, and *glmM*, suggesting coordinated regulation with other cellular processes (4). DacA is essential for viability, as loss of *dacA* function is lethal unless compensatory suppressor mutations arise, indicating that c-di-AMP production is required for cellular homeostasis. YbbR is a membrane protein encoded by the gene immediately downstream of *dacA* and has been implicated in regulating DacA activity (6, 26). Its molecular function remains unclear, although it likely interacts with DacA and modulates its cyclase activity. In contrast, GlmM is a phosphoglucosamine mutase that catalyzes a key step in peptidoglycan precursor synthesis by converting glucosamine-6-phosphate to glucosamine-1-phosphate (8). The structure of this operon with *glmM* and *dacA* within the same operon suggests a functional link between cell wall biosynthesis and c-di-AMP signaling.

DacA requires oligomerization for full enzymatic activity. Studies have shown that GlmM directly interacts with DacA, forming a complex that inhibits cyclase function. GlmM sterically interferes with the DAC domain, limiting access to the active site and preventing the oligomeric assembly required for catalysis. As a result, GlmM suppresses c-di-AMP synthesis by blocking substrate access and disrupting formation of the active oligomeric state (27). This interaction provides a mechanistic link between metabolic flux in cell wall precursor synthesis and second messenger production. The *S. aureus* DacA catalytic domain forms a stable dimer with outward-facing active sites that are not positioned for catalysis, requiring higher-order head-to-head assembly for activity. Consistent with this, multiple DacA dimers are needed for cyclase function. This inhibition is species-selective, as GlmM from gram-negative bacteria does not inhibit *S. aureus* DacA under comparable conditions (26).

This interaction suggests that *S. aureus* coordinates c-di-AMP levels with cell envelope biogenesis. The interaction between DacA and GlmM may couple c-di-AMP synthesis to peptidoglycan precursor availability. This coupling may contribute to envelope integrity and osmotic balance, processes influenced by c-di-AMP signaling. This association between c-di-AMP synthesis and cell envelope metabolism is not unique to *S. aureus*. In *B. subtilis*, CdaA, CdaR, and GlmM form a conserved module, in which CdaR modulates cyclase activity and is functionally connected to potassium homeostasis, a major downstream target of c-di-AMP (27). In *S. aureus*, however, the upstream signals that regulate DacA activity remain unresolved. While GlmM provides a direct metabolic link, the role of YbbR is still unclear, and whether it responds to specific environmental or intracellular cues has not been established. The requirement of DacA depends on growth conditions. *dacA* cannot be deleted in rich medium, but mutants can be obtained in chemically defined medium, and growth can be restored by suppressor mutations in osmolyte and amino acid transporters and in aerobic respiration genes. DacA is also dispensable under anaerobic conditions, linking c-di-AMP synthesis to respiratory and osmotic demands (28). Consistent with a conditional role of YbbR, *ybbR* mutants show increased acid sensitivity that can be tolerated by suppressor mutations, including mutations in *dacA*, with modest changes in c-di-AMP levels (29). Multiple inputs can converge to tune c-di-AMP levels, integrating metabolic state with environmental conditions. In *S. aureus*, DacA is the primary and sole source of c-di-AMP synthesis; therefore, abnormal cellular c-di-AMP levels, including both depletion and accumulation, impair growth, highlighting c-di-AMP homeostasis as a potential target for antibacterial development.

## DEGRADATION PATHWAYS OF c-di-AMP IN *S. AUREUS*

The intracellular level of c-di-AMP in *S. aureus* is balanced not only by synthesis but also by controlled degradation through specific phosphodiesterases (PDEs). These enzymes hydrolyze c-di-AMP to linear 5′-phosphoadenylyl-(3′→5′)-adenosine (pApA), which can then be further broken down into AMP. Three classes of c-di-AMP PDEs have been identified in bacteria: GdpP, Pde2, and PgpH (15, 30). *S. aureus* encodes two of these enzymes: the major phosphodiesterase GdpP and a second enzyme commonly called Pde2 (Fig. 2). In contrast, it lacks a PgpH-type phosphodiesterase, which is found in some other gram-positive bacteria such as *L. monocytogenes* and *B. subtilis* but not in staphylococci (15). GdpP, a c-di-AMP phosphodiesterase, contains an N-terminal PAS sensory domain and a C-terminal DHH/DHHA1 catalytic domain, with the DHH motif conserved among many nucleotide phosphodiesterases. GdpP cleaves c-di-AMP in one step to yield pApA (30). Loss-of-function mutations or deletion of *gdpP* lead to markedly elevated intracellular c-di-AMP, indicating that GdpP is the major c-di-AMP phosphodiesterase in *S. aureus* (29, 31). The *gdpP* gene is co-transcribed with genes encoding ribosomal protein L9 and the DNA replication protein DnaC in a conserved operon, which may suggest that c-di-AMP degradation is coordinated with growth and DNA replication: as the cell prepares for division, expression of GdpP along with ribosomal and replication proteins, may increase to prevent excessive accumulation of c-di-AMP during rapid growth. GdpP’s PAS domain appears to regulate its activity in response to signals; notably, the PAS domain can bind heme, and heme binding inhibits GdpP’s phosphodiesterase activity (32, 33). This finding suggests that heme binding could modulate c-di-AMP degradation by GdpP in *S. aureus*. Because heme binding inhibits GdpP phosphodiesterase activity, heme exposure may promote c-di-AMP accumulation, although the physiological contexts in which this occurs remain unclear. GdpP also contains a degenerate GGDEF domain, normally associated with c-di-GMP synthesis but catalytically inactive here. Mutations in this domain reduce GdpP activity, indicating a regulatory role in c-di-AMP hydrolysis, possibly through ATP binding or structural support.

The second *S. aureus* phosphodiesterase, Pde2, is a cytosolic DHH/DHHA1 family enzyme that preferentially hydrolyzes pApA to AMP. GdpP and Pde2 therefore appear to act sequentially: GdpP converts c-di-AMP to pApA, and Pde2 further degrades pApA to AMP (15). Similar functional partitioning has been reported in other bacteria. In *S. aureus*, however, the regulation of Pde2 remains to be determined. Studies in *L. monocytogenes*, which encodes GdpP/PdeA and PgpH, suggest that distinct phosphodiesterases can respond to different signals and act cooperatively to maintain c-di-AMP homeostasis (13). GdpP and Pde2 in *S. aureus* may therefore be regulated by distinct cellular signals. In addition, pApA itself may act as a regulatory molecule. In some bacteria, pApA inhibits phosphodiesterase activity (29). For example, pApA inhibits *B. subtilis* GdpP, thereby limiting c-di-AMP breakdown through feedback inhibition. In *S. aureus*, accumulated pApA inhibits GdpP activity. Pde2 preferentially hydrolyzes pApA to AMP, thereby preventing pApA accumulation and facilitating continued GdpP-dependent c-di-AMP degradation (29).

Maintaining c-di-AMP homeostasis is critical, as both excess and depletion are detrimental to *S. aureus*. Elevated c-di-AMP, as in *gdpP* mutants, causes reduced cell size, thickened cell walls, and altered osmotic resistance, reflecting dysregulation of c-di-AMP-controlled pathways. In contrast, c-di-AMP depletion through DacA inactivation or phosphodiesterase overexpression severely impairs growth and can lead to cell death. Decreased cellular c-di-AMP can result in uncontrolled potassium uptake, leading to osmotic imbalance and increased turgor pressure. This osmotic stress can compromise cell envelope integrity, thereby contributing to growth defects in *S. aureus* (34). Accordingly, *S. aureus* requires precise control of intracellular c-di-AMP levels. Consistent with this, mutations in phosphodiesterase genes, particularly *gdpP*, have been repeatedly linked to altered antibiotic resistance and stress tolerance. Impaired c-di-AMP degradation alters cell wall homeostasis and osmotic balance, thereby affecting susceptibility to cell envelope-targeting antibiotics such as β-lactams. Accordingly, *gdpP* loss-of-function mutations frequently arise under antibiotic selection in both clinical and laboratory settings, highlighting the importance of c-di-AMP degradation in *S. aureus* adaptation.

## REGULATORY AND PHYSIOLOGICAL ROLES OF c-di-AMP IN *S. AUREUS*

**Fig 3:**
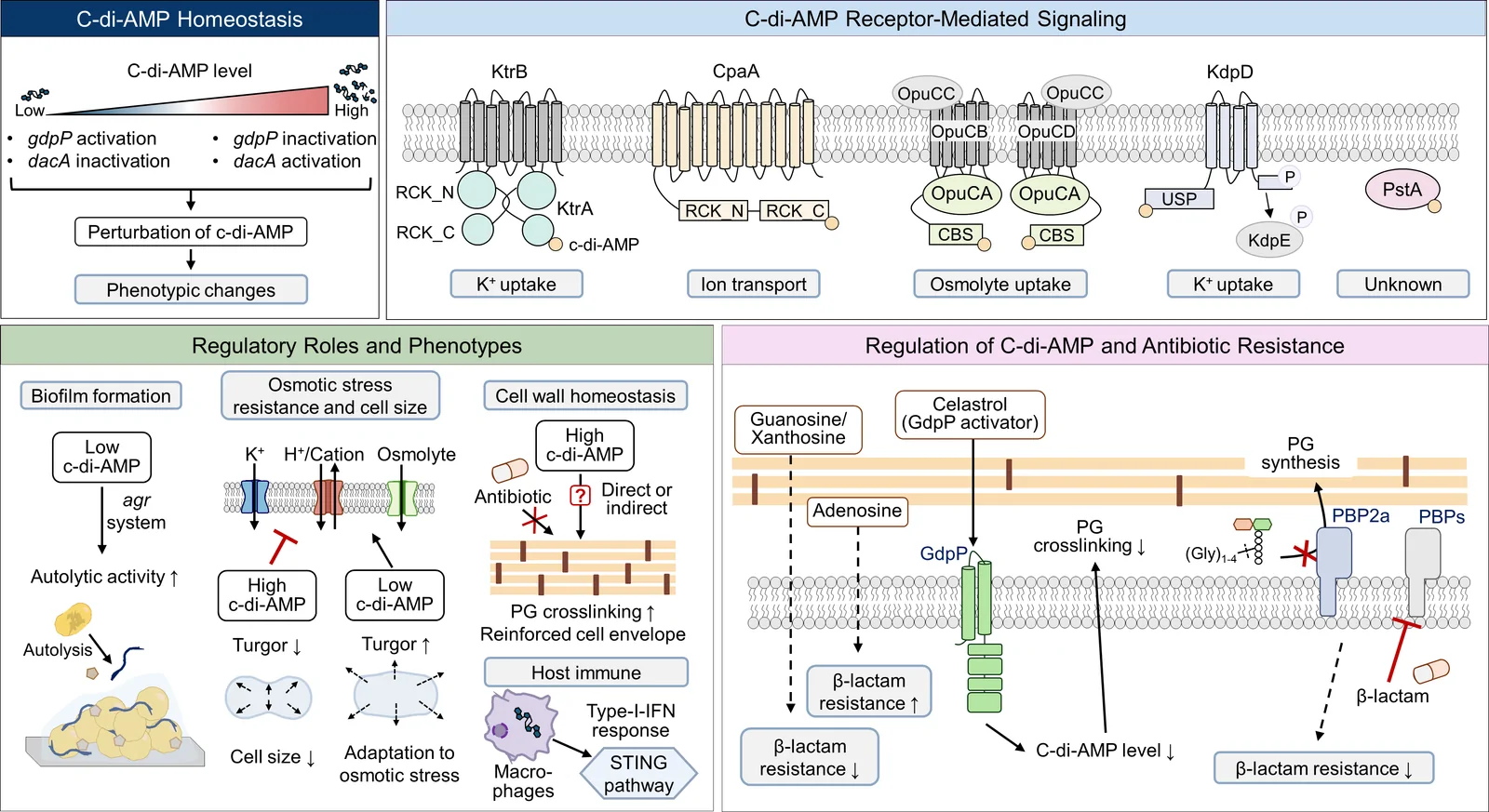
c-di-AMP signaling and cellular responses in *Staphylococcus aureus*. (Top left) Intracellular c-di-AMP levels are controlled by the opposing activities of DacA and GdpP. Reduced c-di-AMP levels are associated with *gdpP* activation or *dacA* inactivation, whereas elevated c-di-AMP levels result from *gdpP* inactivation or *dacA* activation. Perturbation in c-di-AMP homeostasis leads to downstream phenotypic changes, reflecting its central role in bacterial physiology. (Top right) c-di-AMP binds to multiple receptor proteins, directly modulating their activity. c-di-AMP binds to the RCK_C domain of KtrA, regulating KtrB and controlling K^+^ uptake. Similarly, CpaA contains an RCK_C domain that interacts with c-di-AMP to modulate ion transport. For osmolyte uptake, c-di-AMP binds to the CBS domain of OpuCA within the OpuC system, contributing to osmotic homeostasis. In addition, c-di-AMP regulates the KdpDE two-component system, through interaction with the USP domain of KdpD, thereby influencing K^+^ uptake. PstA also binds c-di-AMP, although its physiological role remains unclear. (Bottom left) c-di-AMP levels modulate *agr* activity to control biofilm formation, where low c-di-AMP promotes autolysis and extracellular DNA (eDNA) release, contributing to biofilm formation. c-di-AMP also regulates osmotic stress responses by controlling K^+^, H^+^/cation, and osmolyte transport, thereby affecting turgor pressure and cell size. Elevated c-di-AMP is associated with enhanced peptidoglycan crosslinking and reinforced cell envelope integrity, although the underlying mechanisms remain unclear. Additionally, c-di-AMP released during infection activates STING in macrophages, inducing type I interferons and proinflammatory cytokines that promote tissue inflammation. (Bottom right) c-di-AMP levels are linked to β-lactam resistance through their effects on cell wall synthesis and peptidoglycan crosslinking. Purine nucleosides such as guanosine and xanthosine reduce β-lactam resistance in MRSA, whereas adenosine increases oxacillin resistance. Activation of GdpP by celastrol lowers c-di-AMP levels, leading to decreased peptidoglycan crosslinking and increased amounts of precursors with shortened glycine bridges that cannot be processed by PBP2a, making MRSA susceptible to β-lactam antibiotics.

Beyond its direct interactions with specific receptor proteins, c-di-AMP influences a broad range of physiological processes in *S. aureus*. Intracellular c-di-AMP acts as a global signal that coordinates growth, cell envelope homeostasis, ion transport, osmotic adaptation, and stress responses. These effects are mediated by a defined set of c-di-AMP receptors in *S. aureus*, including KtrA, CpaA, KdpD, PstA, and OpuCA. Through these targets, c-di-AMP links changes in nucleotide levels to specific cellular responses. Perturbation of c-di-AMP homeostasis, whether through accumulation in *gdpP* mutants or depletion caused by impaired DacA function, leads to marked phenotypic changes. Below, key cellular processes controlled by c-di-AMP in *S. aureus* are discussed (Fig. 3).

### Bacterial growth and cell size

c-di-AMP is essential for normal growth in *S. aureus*, and reduced intracellular c-di-AMP causes severe growth defects and can be lethal. This likely reflects its central role in coordinating cell envelope homeostasis and osmotic control. Without proper c-di-AMP signaling, *S. aureus* cannot adequately regulate ion transport, osmolyte balance, and cell wall maintenance, resulting in growth impairment or lysis. In contrast, excess c-di-AMP, as in a *gdpP* deletion mutant, is associated with reduced cell size, compact morphology, and envelope remodeling (31). These phenotypes likely arise from dysregulation of c-di-AMP receptor-mediated pathways. KtrA, the cytoplasmic gating component of the Ktr potassium uptake system, binds c-di-AMP through its RCK_C domain. Structural studies have shown that c-di-AMP binding constrains KtrA in a conformation that suppresses potassium uptake through KtrB (35, 36). Similarly, the cation/proton antiporter CpaA also contains an RCK_C domain that binds c-di-AMP, and this interaction stimulates CpaA-mediated potassium export (35, 37). These targets place c-di-AMP at the center of intracellular ion homeostasis. Because potassium uptake and proton exchange strongly influence turgor and cytoplasmic conditions, dysregulation of these systems readily leads to altered growth and cell morphology. Elevated c-di-AMP is also associated with thicker cell walls, reduced autolysis, and increased peptidoglycan crosslinking. Under these conditions, *S. aureus* appears to reinforce the cell envelope, which may contribute to the reduced cell size phenotype. The importance of this regulatory axis is underscored by the observation that elevated c-di-AMP can rescue otherwise lethal defects such as loss of lipoteichoic acid, presumably through altered osmotic control and enhanced peptidoglycan architecture (31). Thus, c-di-AMP is a central coordinator of growth, linking ion transport and envelope stability to cellular morphology and viability.

### Osmotic stress resistance

One of the clearest functions of c-di-AMP in *S. aureus* is the control of osmotic adaptation. This role is mediated by several receptor proteins: KtrA regulates potassium uptake through the Ktr system, CpaA mediates cation/proton exchange, KdpD controls induction of the high-affinity Kdp potassium transport system, and OpuCA regulates uptake of osmoprotectants such as carnitine through OpuC. These proteins place c-di-AMP at the center of ionic and osmolyte homeostasis. KdpD is particularly important during potassium limitation or osmotic stress. In the KdpDE two-component system, KdpD senses potassium starvation and activates KdpE, which induces expression of the *kdp* transport genes (38). In *S. aureus*, KdpD contains a cytosolic USP domain that binds c-di-AMP. This interaction inhibits KdpD autokinase activity and prevents activation of the Kdp system (35, 39). c-di-AMP therefore suppresses not only basal potassium uptake through KtrA but also inducible high-affinity potassium uptake through KdpD. When c-di-AMP levels are high, potassium accumulation is restrained; when c-di-AMP levels fall, this inhibition is relieved, allowing increased potassium uptake. A similar principle applies to osmoprotectant uptake. OpuCA, the ATPase component of the OpuC transporter, contains tandem CBS domains that bind c-di-AMP. This interaction inhibits ATPase activity and reduces osmolyte import (40). c-di-AMP therefore limits both potassium uptake and compatible solute accumulation, preventing excessive turgor under nonstress conditions. When c-di-AMP levels decline, these uptake systems become more active, supporting adaptation to osmotic stress. Thus, c-di-AMP functions as a central signal of osmotic and ionic sufficiency in *S. aureus*. Through KtrA, CpaA, KdpD, and OpuCA, it exerts multilayered control over osmotic homeostasis.

### Biofilm formation

Unlike c-di-GMP, which often promotes biofilm formation in many bacteria, c-di-AMP appears to suppress biofilm formation in *S. aureus*. Elevated c-di-AMP, such as after *gdpP* inactivation, is often associated with reduced biofilm formation, whereas lower c-di-AMP can promote biofilm development (31). Reduced c-di-AMP is linked to increased release of extracellular DNA and matrix material, which support biofilm formation (41). Although no biofilm-specific c-di-AMP receptor has been identified in *S. aureus*, known receptor proteins likely influence this phenotype indirectly by altering envelope homeostasis, autolysis, and osmotic adaptation. High c-di-AMP suppresses potassium and osmolyte uptake through KtrA, KdpD, and OpuCA, while c-di-AMP also binds the putative cation/proton antiporter CpaA (35, 39, 40). Because controlled autolysis contributes to extracellular DNA release, this envelope-stabilizing effect may help explain the reduction in biofilm formation. c-di-AMP signaling also appears to intersect with broader regulatory pathways such as Agr, which influences biofilm maturation and dispersal (42). Thus, the effects of c-di-AMP receptor-mediated signaling extend beyond ion and osmolyte transport to biofilm-related traits. In *S. aureus*, regulation of biofilm formation by c-di-AMP is likely an indirect consequence of altered envelope physiology and stress adaptation.

### Interplay with host innate immunity

c-di-AMP also contributes to host innate immune activation during *S. aureus* infection. During biofilm formation, bacterial autolysis releases c-di-AMP into the extracellular environment, and macrophage recognition of c-di-AMP through STING induces a type I interferon response (43). Exposure to biofilm-derived c-di-AMP also increased the intracellular survival of *S. aureus* in macrophages, suggesting that STING activation may interfere with bacterial clearance under these conditions (43). During *S. aureus* infection, c-di-AMP production can be influenced by its metabolism. Thymidine starvation in *S. aureus* increases c-di-AMP production, and thymidine-dependent small colony variants induce more STING-dependent inflammation than the parental strain (44). In a mouse lung infection model, these variants increased inflammatory cytokine production and neutrophil recruitment to the airways (44). The macrophage response also depends on the bacterial growth state, as planktonic *S. aureus* induced stronger STING IRF3-dependent IFN-β production than biofilm-associated bacteria (45). These findings indicate that c-di-AMP connects bacterial physiology with host innate immunity, although the outcome of this response may vary with bacterial metabolism and host environments (44).

### Cell wall homeostasis and antibiotic resistance

One of the major roles of c-di-AMP signaling in *S. aureus* is the regulation of cell wall homeostasis and resistance to cell wall-targeting antibiotics. Elevated c-di-AMP is associated with thicker peptidoglycan, increased crosslinking, and reduced autolysis, all of which reinforce the cell envelope. In contrast, reduced c-di-AMP impairs envelope homeostasis and increases sensitivity to cell wall stress (31, 46). Although c-di-AMP does not directly bind the core peptidoglycan synthetic enzymes, its receptor proteins strongly influence cell wall physiology through control of ionic and osmotic balance. Because potassium levels, proton flux, and compatible solutes affect turgor and mechanical stress on the envelope, c-di-AMP-mediated regulation of KtrA, CpaA, KdpD, and OpuCA strongly influences cell wall maintenance. The rescue of lipoteichoic acid-deficient mutants by elevated c-di-AMP further supports the idea that altered osmotic control can partly compensate for envelope defects. These changes are closely linked to antibiotic susceptibility (47, 48). The relationship between c-di-AMP and β-lactam resistance has been extensively investigated. Mutations that increase c-di-AMP, such as loss of GdpP, enhance resistance to β-lactams, whereas mutations that reduce c-di-AMP increase susceptibility (49, 50). In MRSA, elevated c-di-AMP has also been associated with increased β-lactam resistance, including PBP2a-dependent resistance (11, 51). Thus, c-di-AMP appears to promote β-lactam resistance through changes in peptidoglycan structure and autolysis and through effects on PBP2a-dependent resistance. We recently showed that reducing intracellular c-di-AMP levels restores β-lactam susceptibility in MRSA and identified celastrol as a GdpP activator that facilitates c-di-AMP degradation, thereby altering peptidoglycan structure (11). This mechanism of action reduces peptidoglycan crosslinking and changes the glycine bridge length of peptidoglycan precursors. In particular, celastrol treatment leads to a decrease in muropeptides containing full-length (Gly)_5_ bridges while increasing those with shorter (Gly)_1–4_ bridges. Because PBP2a requires (Gly)_5_ containing substrates for efficient crosslinking and is less effective with muropeptides containing shorter glycine bridges, these changes result in resensitization of MRSA to β-lactams (Fig. 3). Thus, GdpP activation may represent a strategy to overcome β-lactam resistance by exploiting the substrate preference of PBP2a. Another c-di-AMP receptor, PstA, may also have a role in c-di-AMP-dependent adaptation to cell wall stress, although its downstream targets remain unclear (35). PstA is a PII-like trimeric protein that binds c-di-AMP with high affinity. As a PII-like protein, PstA may couple c-di-AMP binding to other regulatory pathways, although its downstream function in *S. aureus* remains unclear.

## PERTURBING INTRACELLULAR c-di-AMP BALANCE AS A NOVEL ANTIBACTERIAL TARGET

Given the essentiality of c-di-AMP for *S. aureus* viability and its role in antibiotic resistance, there is considerable interest in targeting c-di-AMP signaling for therapeutic benefit. Disrupting c-di-AMP homeostasis can weaken bacteria or resensitize them to existing antibiotics. Below, we discuss approaches to targeting c-di-AMP homeostasis in *S. aureus*.

### Inhibition of c-di-AMP synthesis by DacA inhibitors

Because DacA is essential in *S. aureus* and many gram positives, small-molecule inhibitors of DacA could have potent antibiotic effects. Inhibiting DacA would acutely lower c-di-AMP, leading to growth arrest and increased susceptibility to stresses. However, designing such inhibitors is challenging due to the need for specificity. Nonetheless, proof-of-concept studies have identified compounds that interfere with DAC activity. For example, a bromophenol derivative, bromophenol thiohydantoin, was reported as a weak inhibitor of *B. subtilis* DisA, the prototype diadenylate cyclase (52). More recently, screening of natural compounds revealed that some polyphenols, such as tannic acid and flavonoids, can inhibit cyclase activity: tannic acid, theaflavin-3′-gallate, and theaflavin-3,3′-digallate showed inhibitory effects on DisA *in vitro*. Tannic acid even inhibited both DisA and a bacterial PDE in that study (53). While these polyphenols are not specific enough for clinical use, they demonstrate that DAC enzymes have druggable pockets. Structure-guided approaches are being used to develop inhibitors that target the active site or disrupt the interaction between DacA and GlmM. Because DacA oligomerization is required for full activity, disrupting the dimer interface could block c-di-AMP synthesis. A molecule that interferes with DacA oligomerization, as GlmM does, could therefore inhibit DacA. Notably, DacA is often difficult to delete genetically, suggesting that chemical inhibition of DacA could be bactericidal. However, effective inhibitors would need to penetrate the bacterial cell and evade efflux, which poses an additional challenge for medicinal chemistry.

### Inhibition of c-di-AMP degradation by PDE inhibitors

Inhibition of c-di-AMP phosphodiesterases such as GdpP would increase intracellular c-di-AMP. Although elevated c-di-AMP is associated with antibiotic resistance, excessive accumulation of this second messenger can also impair bacterial fitness. In *L. monocytogenes*, for example, a double phosphodiesterase mutant with elevated c-di-AMP showed slow growth and reduced virulence in mice (13). Similarly, *S. aureus gdpP* mutants, although more resistant to certain antibiotics, often form smaller colonies and may incur fitness costs (31). Phosphodiesterase inhibition may also sensitize bacteria to particular stresses. For example, increased c-di-AMP could reduce biofilm formation or impair cell division, potentially increasing susceptibility to host defense mechanisms (31). In intracellular infections, elevated bacterial c-di-AMP can also enhance host immune activation and thereby restrict infection (3, 54). One related example is the secreted phosphodiesterase CdnP of group B *Streptococcus*, which degrades c-di-AMP in the host environment; inhibition of such an enzyme could allow extracellular c-di-AMP to accumulate and promote host immune detection (55). Although no specific GdpP inhibitor has yet been reported, the success of phosphodiesterase inhibitors in other systems suggests that bacterial phosphodiesterases may also be druggable. In addition, some polyphenols such as tannic acid inhibit not only DisA but also the *B. subtilis* phosphodiesterase YybT, suggesting that nucleotide-like compounds or allosteric inhibitors could be identified. Phosphodiesterase inhibitors may be most useful as adjuvants rather than stand-alone agents, as elevated c-di-AMP could shift bacteria toward a less virulent state or alter susceptibility to cell wall-active antibiotics, although this approach would require careful control to avoid increasing antibiotic tolerance.

### Synthetic c-di-AMP analogs and competitive inhibitors

Designing c-di-AMP analogs that resist degradation or interfere with receptor signaling could disrupt the c-di-AMP network. For example, a nonhydrolyzable c-di-AMP analog that resists phosphodiesterase cleavage could accumulate in the cell and either overstimulate signaling or occupy receptor binding sites. Alternatively, analogs that bind receptors without inducing the normal conformational response could act as antagonists. Although most work on cyclic dinucleotide analogs has focused on improving stability or STING activation for vaccine and cancer applications, these approaches may also inform antibacterial design. One example is 4′-thio-c-di-AMP, which was reported to act as a potent STING agonist (56). Such analogs might also bind c-di-AMP receptors, although their effects on bacterial signaling remain unclear. Another example is cyclic-di-IMP, which could potentially interact with c-di-AMP receptors, although its effects on receptor signaling remain unclear. In addition, the DacA active site could be targeted with ATP analogs or other compounds that occupy the nucleotide binding pocket and inhibit c-di-AMP synthesis. Structure-guided and computational approaches have begun to explore inhibitors that bind the active site of CdaA/DacA. Compounds that disrupt c-di-AMP signaling could impair bacterial growth or stress adaptation.

### Exploiting metabolic links for purine pathway modulators

Recent studies suggest that c-di-AMP can also be modulated indirectly through central metabolism, particularly purine homeostasis. Because ATP is the substrate for c-di-AMP synthesis and guanine nucleotide pools influence related signaling pathways such as (p)ppGpp, changes in purine metabolism can alter c-di-AMP levels and antibiotic susceptibility. A notable example is the finding that exogenous guanosine and xanthosine lower c-di-AMP levels in *S. aureus* and restore β-lactam susceptibility in MRSA, whereas adenosine has the opposite effect and increases oxacillin resistance (57). These observations indicate that purine flux influences c-di-AMP-dependent regulation. Mechanistically, guanosine may alter the balance of ATP and GTP pools, affect alarmone metabolism, or influence GdpP-dependent turnover, thereby reducing intracellular c-di-AMP. Consistent with this, mutations in purine transport and salvage pathways affect β-lactam resistance, and the adjuvant effect of guanosine depends on intact c-di-AMP regulation (Fig. 3) (11, 57). This suggests that antibiotic susceptibility might be restored without directly targeting DacA or GdpP. Instead, purine nucleosides, purine analogs, or inhibitors of purine biosynthesis could be used to shift c-di-AMP homeostasis and enhance the activity of conventional antibiotics. These findings highlight the integration of c-di-AMP signaling with core metabolism and suggest that indirect modulation of this pathway may provide a useful route for combination therapy.

Overall, current studies support c-di-AMP signaling as a promising antibacterial vulnerability in *S. aureus*. Direct inhibition of c-di-AMP synthesis, modulation of phosphodiesterase activity, use of synthetic cyclic dinucleotide analogs, and indirect metabolic perturbation through purine pathways each represent distinct approaches to manipulate this pathway. Although substantial challenges remain, recent findings indicate that c-di-AMP homeostasis is not only central to *S. aureus* physiology but also increasingly tractable as a target for antimicrobial intervention.

## CONCLUSION

c-di-AMP has emerged as a central regulatory nucleotide in *S. aureus*, connecting cell envelope physiology, osmotic balance, ion transport, stress adaptation, and antibiotic resistance. Unlike many signaling pathways that control a narrow set of outputs, c-di-AMP functions as a broader homeostatic signal. Its importance is reflected by the fact that both insufficient and excessive levels are detrimental to bacterial growth. Although several key components of this pathway have been identified, how *S. aureus* senses environmental or metabolic cues and converts them into changes in c-di-AMP levels remains incompletely understood. Recent studies have increasingly recognized the link between c-di-AMP regulation and cell envelope remodeling in *S. aureus*. Elevated c-di-AMP levels are associated with reduced cell size, altered peptidoglycan architecture, reduced autolysis, and increased resistance to β-lactam antibiotics, effects that appear to arise largely through regulation of ion and osmolyte transport systems. These findings suggest that altered c-di-AMP levels can influence antibiotic susceptibility, biofilm formation, and fitness under stress conditions.

c-di-AMP signaling represents an attractive but challenging target. Direct inhibition of c-di-AMP production could lower c-di-AMP levels below the threshold required for normal growth, potentially leading to bacterial killing or enhanced sensitivity to cell wall-targeting antibiotics. In contrast, inhibition of c-di-AMP degradation would increase c-di-AMP levels, which may impose fitness costs in the host environment. However, elevated c-di-AMP can also promote β-lactam resistance, indicating that this approach requires careful consideration. Recent findings that purine nucleosides can alter c-di-AMP levels and restore β-lactam susceptibility in MRSA further suggest that metabolic modulation can indirectly affect this pathway. However, the upstream signals that regulate c-di-AMP production are still not fully defined. How c-di-AMP leads to cell wall remodeling, and which effector proteins are involved in this regulation, also require further clarification. In addition, it remains important to understand how c-di-AMP signaling operates during infection, antibiotic exposure, and host-associated stress. c-di-AMP is not only an essential signaling molecule but also a regulatory hub that allows *S. aureus* to maintain envelope integrity while adapting to changing environmental conditions. This pathway may provide new insight into bacterial homeostasis and the development of strategies against *S. aureus* by disturbing its ability to balance growth, stress resistance, and cell wall integrity.
